# Mitigating the Environmental Impacts from Pig and Broiler Chicken Productions: Case Study on a Citrus Extract Feed Additive

**DOI:** 10.3390/ani13233702

**Published:** 2023-11-29

**Authors:** Hoa Bui, Sekhou Hedaly Cisse, Mathilde Ceccaldi, Aurélie Perrin, Mohammed El Amine Benarbia, Pierre Chicoteau

**Affiliations:** 1 Nor-Feed SAS, 3 Rue Amedeo Avogadro, 49070 Beaucouzé, France; sekhou.cisse@norfeed.net (S.H.C.); amine.benarbia@norfeed.net (M.E.A.B.); pierre.chicoteau@norfeed.net (P.C.); 2 Labcom FeedInTech, 42 Rue Georges Morel, 49070 Beaucouzé, France; 3 EVEA, 11 Rue Voltaire, 44000 Nantes, France; m.ceccaldi@evea-conseil.com (M.C.); a.perrin@evea-conseil.com (A.P.)

**Keywords:** life-cycle assessment, citrus extract, broiler chicken, swine, feed additives

## Abstract

**Simple Summary:**

Mitigating environmental footprint is one of the most important pillars for building sustainable livestock production. Evidently, using natural feed additives exploited from agro-food byproducts to optimize feed efficiency and animal performance can bring multiple advantages to the environment. Our study using a life-cycle assessment of a commercial citrus extract feed additive showed that the impacts generated by this additive manufacturing are minor, in comparation to the reduction in environmental issues from pig and broiler chicken productions, thanks to the additive’s effects on improving animal growth performances. More precisely, the manufacturing and distribution of a 25 kg bag of citrus extract feed additive potentially emits 13.1 kg of CO_2_ equivalent; uses 5.3 m^2^ of land year round; and consumes 66 L of water. Meanwhile, using one bag of 25 kg citrus extract feed additive in feed at a low inclusion rate (250 g per ton of completed feed) can reduce CO_2_ equivalent emission by 5 and 6 tons and water consumption by 82 m^3^ and 201 m^3^ in swine and broiler productions, respectively. In parallel, land surface used is saved by 7000 m^2^ year round in both mentioned livestock systems. Such results demonstrate the great interest in using citrus extract feed additive as an additional tool for mitigating environmental impacts from livestock farming activities.

**Abstract:**

The rapid expansion of the livestock production sector to meet the world population’s demand is posing a big challenge to environmental sustainability. Plant-based feed additives extracted from agro-food byproducts could potentially result in multiple outcomes: reducing food-processing wastes and improving animal growth performances, hence mitigating environmental impacts of meat production chains. This presented study was carried out to assess the environmental impacts of the use of a commercial citrus extract feed additive (CEFA) in swine and broiler chicken farming. Life-cycle assessment (LCA) was applied to assess the impact of manufacturing and distributing one 25 kg bag of CEFA and its use in feed in broiler chicken and swine productions. With regards to CEFA manufacturing and distribution, results showed that most of the impact came from the production of CEFA ingredients, accounting for 70% of the impact generated. The remaining 30% effect was divided between transportation to the customer (25%), CEFA packaging (3%), and CEFA manufacturing and production loss (2%). When enlarging the scope, the use of the CEFA in pigs and broilers’ diets was shown to improve the measured environmental indicators, compared to such standard systems. Indeed, CEFA-added feeds have demonstrated enhanced growth performances, hence reducing the required amount of consumed feed to achieve the same level of growth. Consequently, this helped reduce environmental issues from animal feed ingredients’ agriculture. To be more specific, the use of one 25 kg bag of CEFA in feed at 250 g per ton of feed led to a reduction of 6 tons of CO_2_ equivalent (CO_2_ eq) emitted along the life cycle of poultry production and 5 tons in the case of fattening pigs. The inclusion of this CEFA in the diet also led to a reduction in the land use footprint by 0.7 hectares and reductions in water consumption by 201 m^3^ and 82 m^3^ for broiler chicken and swine production, respectively. The environmental performance assessment thus showed the interest in using this CEFA in swine and broiler chicken diets to mitigate the environmental impacts.

## 1. Introduction

The animal production sector is one of the biggest users of freshwater and agricultural resources, which account for approximately 70% of water and 30% of ice-free surface land use worldwide [1]. Additionally, this sector is responsible for 18% of the total greenhouse gas generated by human activities [1]. Associated with pigs and broiler chicken production chains, feed-producing activities were demonstrated to be a major source of greenhouse gas emissions at approximately 70% [2]. Consequently, with its predicted rapid growth in the coming decades [3], livestock production indeed causes published concerns about environmental impacts.

The circumstance has raised a huge challenge to the livestock stakeholders to work towards resource-effective production systems and sustainable development strategies. Amongst implemented solutions, using feed additives to improve growth performances could indirectly result in optimizing input resources, as well as mitigating pollution. This approach has been proven by many studies worldwide on different species, so it is considered one of the promising applications for the long term [4]. Potentially, the benefits could be multiplied in the case of natural feed additives, which are exploited from plant-origin or food-processing byproducts, thanks to it reducing waste and adding value to the agro-food production chain. Citrus extract feed additive (CEFA) is, therefore, one good example. Several studies worldwide have been conducted on this food byproduct, used as a dietary additive, which demonstrated its benefits on growth performances in broiler chickens [5,6,7] and swine [8,9,10]. However, to the best of our knowledge, the evaluation of its effects on animal growth performances combined with its environmental impacts has scarcely been studied before.

This study was conducted to assess the environmental footprint of a commercial CEFA (Nor-Spice AB^®^, Nor-Feed SAS), which was characterized and standardized with its active compounds and was proven for its efficiency in improving broiler chickens and swine growth performances. To do so, the life-cycle assessment of the CEFA was carried out according to ISO 14,040 [11] and 14,044 standards [12].

## 2. Materials and Methods

The study was conducted using the life-cycle assessment (LCA) methodology as described in ISO 14,040 [11] and ISO 14,044 [12] standards. LCA is a method to assess the environmental impacts of a product or a service by quantifying the resources used and the emissions to the environment at several stages of its life cycle. The method combines several impact categories that allow the identification of the potential pollution transfer between impact categories. LCA comprises four phases: goal and scope definition, inventory analysis, impact assessment, and interpretation.

### 2.1. Goal and Scope

**Figure 1:**
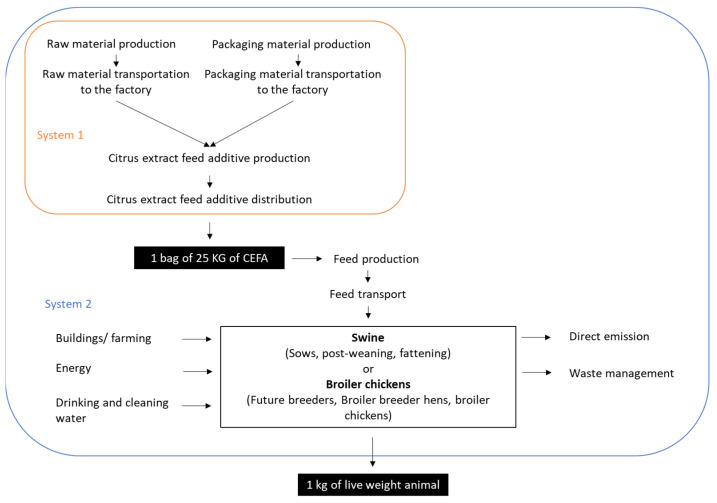
Overview of the system boundary and functional units.

The objective of the study was to assess the environmental impacts of CEFA from the production stage to its use at farms. To this end, two systems were defined, as described in Figure 1.

System 1 represented the production stage of CEFA, in which the production of its ingredients and transportation to an additive-processing plant were included. Additionally, System 1 also consisted of packaging and distributing once CEFA made it to the final customer. In order to assess, objectively, the overall impact of CEFA addition in feed, the using phase in farms was also studied in comparison to animal production without using the additive. Two types of livestock farming were tested: broiler chicken and swine.

System 2 included the production of CEFA-added feed and all farming activities associated with animal production at the farm level.

Two functional units were determined according to each described system. The first functional unit was “produce and distribute one 25 kg bag of CEFA”. For this functional unit, two distribution scenarios were studied: a distribution scenario in France (country of production) and a distribution scenario in Thailand (major customer). The second functional unit was “produce 1 kg of live animal feed with (vs. without) CEFA” in France and in Thailand.

### 2.2. Inventory Analysis

Generic data and specific data were combined to obtain inventories, as described in Table 1.

For System 1, specific data from the company were used to describe the type and quantities of ingredients, quantities of energy, and type of packaging used for the production of the additive (Table 2). Background data related to the production of ingredients and packaging, the transporting vehicles and infrastructures, and the production and delivery of the energy used were obtained from AGRIBALYSE v3.0.1 (2020) and Ecoinvent v3.6 (2020). When it came to lemon extract, this ingredient was produced in Spain. However, due to the lack of specific data to model the lemon extraction process, data for tomato paste (AGRYBALYSE V3.0) were adapted with a specific transformation yield of 6%. For orange peels, Spanish oranges (Ecoinvent) were used instead of Moroccan oranges, since there were no specific data available. Additionally, transformation processes of the orange peel, including peeling and drying, were neglected. A ratio of juice to peel of 40% was applied to set the allocations, based on the AGRIBALYSE database and according to several studies found in literature [13,14].

Production at the Nor-Feed plant in France was modeled using specific data provided from 2019 (Table 3), considering a total production of 1,025,000 kg (CEFA included).

Packaging of the finished product was modeled using specific data for one 25 kg bag of CEFA (Table 4).

The distance of packaging transportation to the Nor-Feed plant was counted for 1450 km, while the distances of CEFA’s ingredients delivery and final product distribution were estimated using Searates (https://www.searates.com/fr/, accessed on 17 June 2020). Finally, all the data used for transports were calculated based on lorry, EURO5, and 16–32 tons (Ecoinvent 3.8).

On the subject of system 2, AGRIBALYSE, a reference database of environmental impact indicators for agricultural products produced in France, was used as the reference for the comparison and adapted to represent performances obtained with the use of the additive using the MEANS InOut software, which implements recommended methodologies defined for the AGRIBALYSE program [15].

Growth performances of CEFA given to animals were obtained from the meta-analysis of trial data conducted at different life stages of animal breeding [16]. Concerning broiler chicken production, the meta-analysis was composed of 9 trials performed in commercial or experimental farms in European countries, Canada, Taiwan, and India. Animals were fed *ad libitum* with commercial feed containing 250 g/T of CEFA during the entire production period. The AGRIBALYSE inventory for the conventional national average of broiler production was used as the reference. Growth performance changes resulting from the use of the additive were implemented at the fattening stage for one parameter (Table 5). The weight of the broiler chicken was adapted to reach trial data on the average daily gain (ADG) and feed conversion ratio (FCR).

In the case of pig production, the meta-analysis was composed of 10 trials performed in Denmark, The United Kingdom, Canada, and Switzerland. Animals were fed *ad libitum* with commercial feed containing 250 g of CEFA per ton of feed during the post-weaned period. The AGRIBALYSE® inventory for the conventional national average of pig production was used as the reference. Growth performance changes resulting from the inclusion of CEFA in feed were implemented at the post-weaned stage and the pig-finishing stage for two parameters (Table 6). The weight of the fattened pig was adapted using trial data on ADG, and the number of days at the post-weaned and finishing stages was adapted to reach trial data on the ADG and FCR.

Data from the meta-analysis used in this study were provided from trials that were carried out in strict accordance with the recommendations set out in the European guidelines for accommodation and care of animals (Directive 86/609/CEE).

### 2.3. Life-Cycle Impact Assessment (LCIA)

The impact assessment was performed using the environmental footprint method (version 2.0) [17], as recommended by the European commission for environmental labeling. A total of 3 indicators over the 16 were selected for being the most representative of the main contributors to the environmental impact of the two studied functions: climate change, land occupation, and water consumption. The 3 selected indicators are described in Table 7.

## 3. Results

### 3.1. Environmental Impacts within Functional Unit 1: Producing and Distributing One 25 kg Bag of CEFA

**Figure 2:**
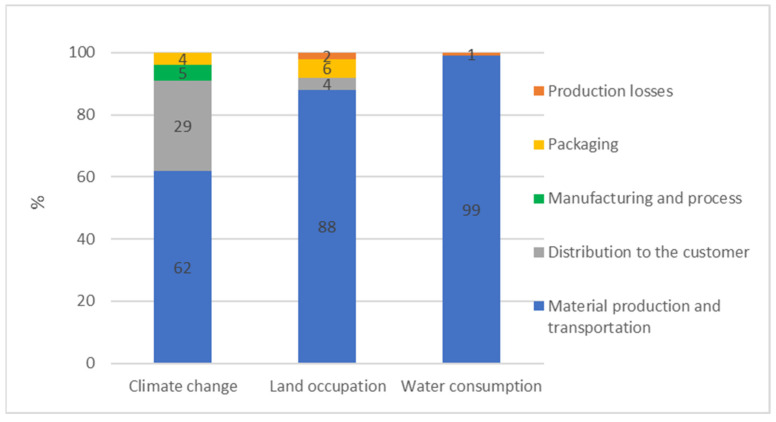
Environmental impacts of producing and distributing one 25 kg bag of CEFA.

In terms of climate change, LCA analysis showed that the entire processing and distribution of one 25 kg bag of CEFA generated 13.1 kg of CO_2_ eq (Figure 2), in which up to 8.12 kg of CO_2_ eq was emitted during the stage of ingredients production and transportation to the Nor-Feed plant. Additionally, distributing the product to domestic and international customers resulted in the emission of 3.78 kg of CO_2_ eq, while manufacturing and packaging processes to transform the raw materials into the final product emitted 1.18 kg of CO_2_ eq. A similar pattern was observed in the case of land occupation (Figure 2). The land surface used for the production of one 25 kg bag of CEFA along its life cycle was estimated at 5.3 m^2^ of arable land year round, which was mainly caused by the land used for citrus agriculture (4.7 m^2^). The remaining 0.6 m^2^ resulted from the packaging (6%), the distribution to customers (4%), and production losses (2%). When it came to water consumption, amongst a total of 66 L of water consumed for producing one 25 kg bag of this CEFA, almost all of it was spent for the production and transportation of CEFA ingredients to the additive processor (Figure 2).

### 3.2. Focus on Environmental Impact of CEFA Production and Transportation to the Factory

**Figure 3:**
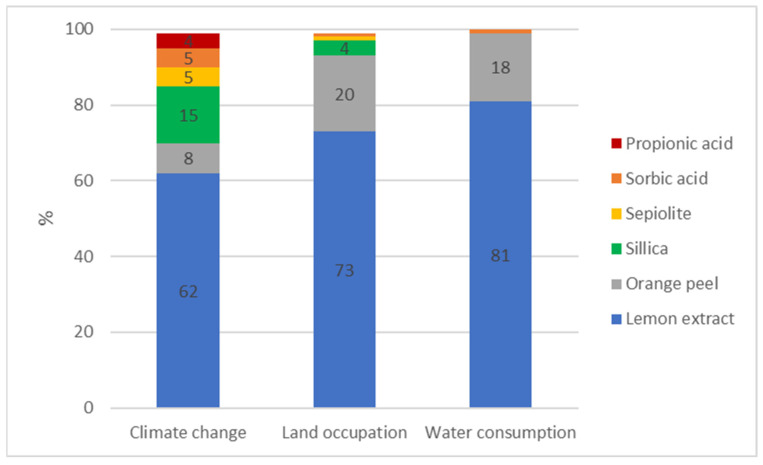
CEFA ingredients’ environmental impacts on climate change, land occupation, and water consumption.

CEFA ingredients production and transportation to the factory represented, on average, 83% of the environmental impact on the three selected indicators. As lemon extract occupies an important proportion in the composition of CEFA, it had a considerable impact, compared to the other remaining raw materials (Figure 3).

Indeed, lemon extract represented more than 50% of CEFA formulation and generated 81% impact on water use, 73% impact on land occupation, and 62% impact on climate change of the total contribution of all ingredients. The percentage of lemon extract in CEFA formulation was therefore lower than the average impact contribution in the three selected indicators. Agricultural activities like fertilization and irrigation were the main contributors, while the transformation process of citrus fruit only represented a minor contributor.

It is important to take into account that lemon extract is a byproduct from juice industries. Because of the lack of data, a proxy was used to represent the transformation process for obtaining lemon extract from fruits. Furthermore, only 6% of this transformation process was allocated to the lemon extract, the main ingredient of CEFA. This allocation was based on mass, and other co-products considered were lemon juice and essential oil. To test the sensitivity of this parameter, we also analyzed the environmental impact of CEFA when 100% of the lemon extract process was allocated to the CEFA.

**Figure 4:**
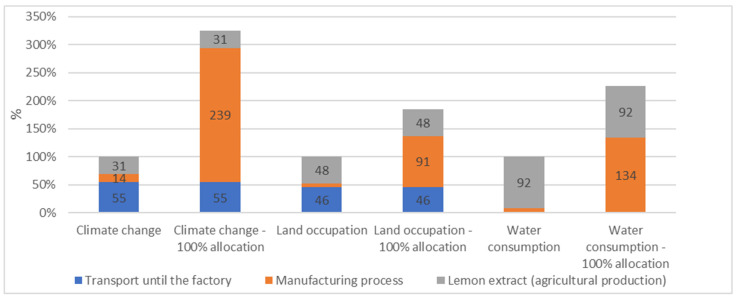
CEFA environmental impact when 100% of the lemon extract and citrus peel transformation process was allocated to the CEFA.

Results showed that the impact of 100% of the lemon extract and citrus peel CEFA on climate change was multiplied by 3, while the impact on water consumption was multiplied by 2 when allocating 100% of the lemon extract transformation process to the CEFA (Figure 4). By contrast, fewer changes were observed on land occupation (Figure 4).

### 3.3. Focus on Environmental Impact of CEFA Distribution to the Final Customer

According to the results presented in Figure 2, CEFA’s distribution to the final customer accounted for 11% of the three environmental impacts, on average, on the three selected indicators. The main environmental impact was observed on climate change, for which the distribution to the final customer accounted for 29%.

**Figure 5:**
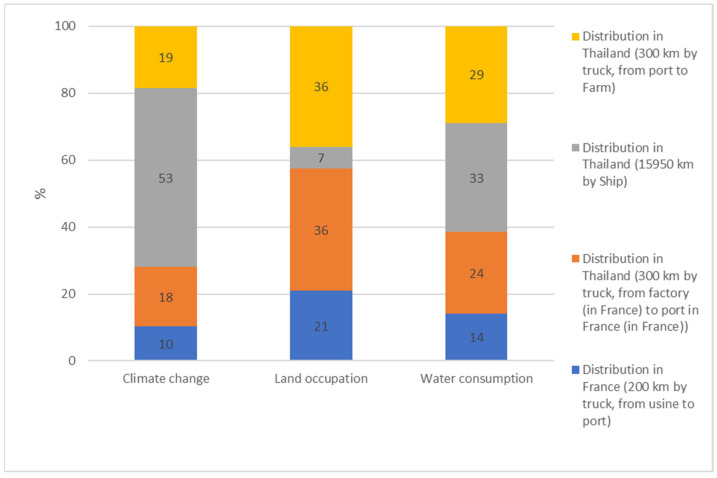
CEFA distribution environmental impacts on climate change, land occupation, and water consumption.

It is important to note that the proportion of CEFA distributed in France (46.5%) and Thailand (53.5%) was equivalent. The exportation of CEFA to Thailand generated the main effect, up to 85% of the overall effect (Figure 5), compared to the distribution in France (15%). In terms of distance, CEFA transportation by truck in France and Thailand represented only a small distance (600 km), compared to transportation by ship (15,950 km). However, its effect on environmental indicators was not negligeable. Indeed, CEFA transportation by truck in France and Thailand accounted for 93.4% of the total environmental impact of CEFA distribution to the final customer on land occupation. It also caused big impacts on the total environmental impact of CEFA distribution to the final customer regarding water consumption (67.4%) and climate change (46.6%).

### 3.4. Environmental Impacts within Functional Unit 2: Manufacturing, Distribution, and Utilization of One 25 kg Bag of CEFA in Farm

**Figure 6:**
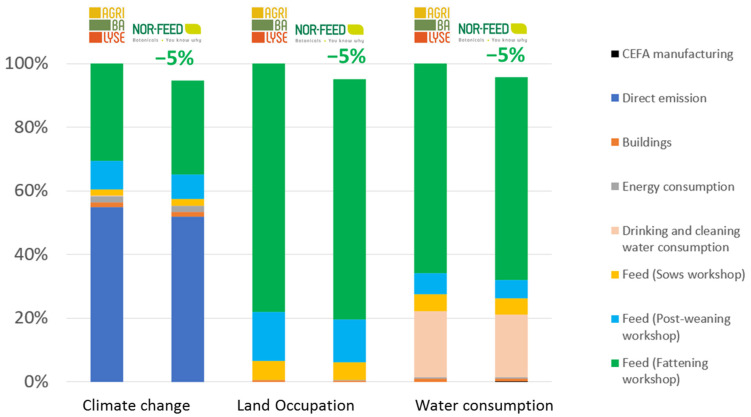
Environmental gains due to the use of CEFA in pig production.

**Figure 7:**
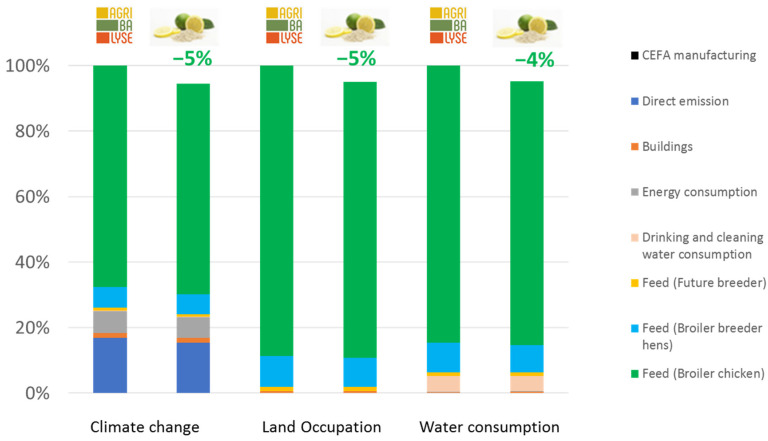
Environmental gains due to the use of CEFA in broiler chicken production.

The environmental benefits that resulted from the use of the CEFA in pigs and broiler chicken productions compared to the standard condition are shown in Figure 6 and Figure 7.

The environmental gains in both production systems fed with CEFA resulted mainly from the reduction in the quantity of feed consumed to produce the same amount of animal live weight. With the CEFA addition, the growth performances were improved, which reduced the husbandry duration, compared to the standard condition. As a result, the emissions related to husbandry, including manure emission and enteric fermentation, were also reduced, especially for swine production. CEFA reduced climate change by approximatively 5% in both broiler chicken and swine productions. More precisely, each 25 kg bag of citrus extract feed additive helped reduce 6 tons of CO2 eq emitted from broiler production and 5 tons in the case of pig production, compared to the same situation without CEFA. In terms of land occupation, every 25 kg of CEFA saved 7000 m^2^ of land used for both studied production systems. The use of 25 kg of CEFA also benefited water consumption, saving up to 201 m^3^ (4%) and 82 m^3^ (5%) of water consumption in boiler and pig productions, respectively.

## 4. Discussion

As mentioned, animal production is one of the most influential sectors on the environment. For more sustainability, it is crucial to evaluate and reduce both the emission of greenhouse gases and the resources needed for their production. To do so, the life-cycle assessment is a good tool to evaluate the environmental footprint, with the aim of identifying areas for improvement to reduce the environmental impact.

The aim of this study was to evaluate the environmental footprint of a citrus extract feed additive in animal nutrition. The main results showed that, for the production and distribution part of the product in itself, the environmental impact of producing and manufacturing one 25 kg bag generated 13.1 kg of CO_2_ eq and required 66 L of water and 5.3 m^2^ of year-round land surface. This is mainly due to the fact that its principal raw material (lemon extract) is a co-product from juice industries. Thus, only 6% of the transformation process of citrus fruit was allocated to the lemon extract that was estimated according to the flow chart of lemon extract production. However, this estimate may constitute a bias on the environmental footprint of CEFA. The use of byproducts of the agro-industry is indeed only starting, as more and more interest is being drawn to this mass considered to be waste (and needed to be treated as such with associated environmental costs) [13,18]. Whilst many studies have recently been conducted on the potential bio-activities of such byproducts, the industrialization of their valorization is only at its debut, meaning that data on the allocation of resources to produce them from the overall resources required for the production of the main crop (here, lemon juice) are scarce. Adding to this, byproducts identified for their potential can represent various plant part origins (e.g., citrus peel, grape pomace [19], or a byproduct from the industrial process (e.g., olive margins [20])), thus increasing the difficulty of allocating resources for the overall process.

In the present study, to compensate for the lack of specific data on lemon extract regarding energy consumption and co-products, the tomato process was used as a proxy to model the extraction process of lemon extract. To evaluate the effect of this choice, a comparison with a distorted allocation, where CEFA would be considered the main crop object and lemon juice was not considered in the equation, was carried out with 100% allocation for CEFA. Our results showed that the environmental impact of producing and distributing one 25 kg bag of CEFA was, in this case, multiplied by 2 or 3, depending on the indicators. Whilst these may appear as huge changes, they still remain negligeable when considering the use of CEFA in system 2 for both swine and broiler chicken productions. The lack of a specific reference in lemon extract regarding energy consumption and co-products use might thus induce uncertainties in the final results, but these aspects remain minor, compared to the environmental effects on the overall use of the product.

Regarding transportation, our study revealed that environmental impacts generated by truck transport were significant, even though it covered only a short distance of the whole distributing chain. It has long been known that truck shipment has a higher environmental footprint than other transportation solutions [21], and this could be a considerable step where the environmental impact of CEFA producing and distributing could be improved. Environmentally responsible alternatives to truck transportation could be used, such as transportation via train freight, where this solution is developed. Future technological developments and their adoption, such as hybrid or full-electric vehicles or more stringent policies on emissions, might also have a positive effect in the future on the life-cycle assessment of CEFA. Moreover, the impact of sea transportation of CEFA was 53.4% on the climate change factor. It is noteworthy to take into consideration the current technological development for the reduction in the emissions of ships that could lead to a further reduction in this important step.

Regarding the use of CEFA in broiler or swine farms, generic data used a reference (AGRIBALYSE®) that showed that the main factors responsible for environmental impacts are feed consumption and direct emission from farming. Our results showed that using CEFA in broiler or swine farms provided benefits to the three environmental threats identified for animal production (climate change, land occupation, and water consumption), compared to standard farming. As shown in the model, since feed consumption represented more than 75% of the environmental impact of swine and broiler chicken production on land occupation and water consumption, as well as for climate change in broiler chickens, it is clear that the improvement of feed efficiency is a key to reducing the environmental impact of livestock farming. Thus, the beneficial impact of CEFA on the reduction in the feed conversion rate (−3% and −7% for broiler chickens and swine productions, respectively) explains, in large part, its beneficial environmental impact. Additionally, in this study, we considered that there was no difference in effluent emissions per day between the CEFA-included situation and the standard situation (only the duration varied). However, it has been generally observed that an improvement in FCR translates to a better use of the diet, explained by the higher uptake of nutrients such as protein and energy. This improved uptake then tends to translate into a reduction in the ammonia load in the farm effluents [22]. It would thus be interesting to investigate the influence of CEFA addition on the quantity and characteristics of farm effluents to assess if this aspect could also further benefit its overall environmental effects. Finally, no variation was considered in the mortality rate; however, several studies have shown the influence of feed quality on this parameter [23]. While it is difficult to implement in a model due to its variability (linked to production stage, climate, farming practices, pathogen pressure…), this represents another important aspect of the environmental impact. Since dead animals do consume feed before they die, this also impacts the overall calculations. Further models should be developed in order to take this complex aspect into consideration.

As shown in Figure 5 and Figure 6, the environmental impact of producing and distributing CEFA was minimal, compared to the environmental impact of the pig and broiler chicken production systems. This may be explained by the low inclusion rate of CEFA in feed (250 g/T) but also the low environmental footprint of producing and distributing CEFA to the final customer. CEFA does indeed represent a double benefit in that it is a byproduct and thus has a reduced environmental impact, compared to solutions that are developed from a main crop, and its effect at the farm level leads to a reduction in the environmental load of the production.

According to literature, many studies showed that additives used in animal nutrition, such as CEFA, can help to positively improve the environmental footprint of the animal production sector, thanks to the benefits they confer on productivity. Blonk and collaborators observed that the general use of feed additives can lead to up to a 10% improvement in the environmental footprint, due to the feed additive effect on productivity and greenhouse gas emissions [24]. Comparatively, in the present study, the use of CEFA alone allowed us to achieve a 5% reduction in these parameters. While this is not as high as the numbers of Blonk et al., it highlights that the choice of the additives used can be crucial, as some may be more potent in their environmental impacts than others. As discussed previously, the use of a natural byproduct of the agro-industry, such as CEFA, might be of high interest in this case, due to the lower impact.

## 5. Conclusions

The environmental impact of producing citrus extract feed additive was mainly linked to raw ingredient production and transportation to the factory, representing 70% out of three selected indicators. The manufacturing and distribution of one 25 kg bag of CEFA potentially emitted 13.1 kg of CO_2_ eq while using 5.3 m^2^ of land year round and consuming 66 L of water. In animal production, the use of one 25 kg bag of CEFA reduced CO_2_ emissions by 5 and 6 tons in swine and broiler chicken, respectively. On the other hand, it reduced land surface use needs by 7000 m^2^ in both broiler chicken and swine productions. In addition, water consumption was saved up to 82 m^3^ and 201 m^3^ in swine and broiler production, respectively. This results from the improving effect of CEFA inclusion in swine and broiler chicken production, which allows ADG to increase by 10.9% and 4.3% and the FCR to reduce by 7.1% and 2.4% in swine and broiler chicken, respectively. Other benefits, such as the reduction in the mortality rate and the modification of effluents, were not included in this study, suggesting a higher impact reduction. These results showed the great interest in CEFA as an additional tool for mitigating livestock farming environmental impacts.

## Figures and Tables

**Table 1:** Origin of data.

Step	Specific Data from Nor-Feed	Generic Data from AGRIBALYSE and Ecoinvent
CEFA ingredients production and transport	Quantity of each ingredient in the formula of the product Origin of each ingredient Type of production of each ingredient	Ingredients production data Ingredients transport data
CEFA packaging production and transport	Quantity of each material of the packaging Origin of each material	Packaging materials production data Packaging materials transport data
CEFA production	Electricity use at the factory Water use at the factory Losses at the factory (and type of management: spreading) Packaging waste produced by the factory Wastewater produced by the factory	Electricity production data Water production data Waste management data Wastewater management data
CEFA distribution	Distances for distribution and transportation means	Transport data
CEFA use	Growth performance of animal production fed with Nor-spice AB (feed conversion ratio and average daily gain)	Animal production data (broiler and pig)

**Table 2:** List of ingredients used for CEFA production.

Ingredient	Used Data	Origin	Transport
Lemon extract	Raw material: lemon {ES} (Ecoinvent 3.5) Processing process approximated by that of tomato concentrate production (AGRIBALYSE V3) Mass allocation based on the ‘lemon extract’ process yield (6%) applied to the raw material and process	Spain	1400 km by truck
Orange peels	Raw material: orange, fresh {ES} (Ecoinvent 3.5) Neglected process (peeling and sun drying) Mass allocation based on juice/peel ratio (40%) AGRIBALYSE V3.0	Morocco	340 km by truck 2200 km by ship
Silica	Raw material: silica sand {DE} (Ecoinvent 3.5)	Spain	830 km by truck
Sepiolite	Raw material: clay {CH} (Ecoinvent 3.5)	Spain	1050 km by truck
Sorbic acid	Raw material approximated by acetic acid {RER} (Ecoinvent 3.5)	Germany	490 km by truck
Propionic acid		France	77 km by truck

**Table 3:** Specific data from the Nor-Feed plant (2019) used for production modeling.

Item	Qt/year	Data Used for Modeling
Electricity consumption	60,000 kWh	Mix French Medium Voltage {FR}
Water consumption	50 m^3^	Drinking water {RER}
Cardboard waste	5900 kg	Average end-of-life hypothesis in France: 70% recycled, 30% not recycled (of which 64% was incinerated with energy recovery, and 36% was buried)
Plastic waste	17,260 kg	Average end-of-life hypothesis in France: 100% not recycled (of which 64% was incinerated with energy recovery and 36% was buried)
Waste water	50 m^3^	Sewer Proxy: potato starch production wastewater
Material losses	1%	Industrial composting

**Table 4:** Data used for packaging model.

Material	Mass/Unit (Bag of 25 kg)	Data Used	Transport	End of Life
Sheet 1: 90% Paper	90 g × 90% =81 g	Coated paper {RER}	1450 km By truck	Average end-of-life hypothesis in France: For the paper part: 70% recycled, 30% not recycled (of which 64% was incinerated with energy recovery, and 36% was buried) For the PE part: 100% not recycled (of which 64% was incinerated with energy recovery, and 36% was buried) Average end-of-life hypothesis in Thailand: For the paper part: 50% recycled, 50% not recycled (of which 6% was incinerated with energy recovery, and 94% was buried) For the PE part: 100% not recycled (of which 6% was incinerated with energy recovery, and 94% was buried)
Sheet 1: 10% polyethylene (PE)	90 g × 10% =9 g	low density polyethylene (LDPE) {GLO}		
Sheet 2: Paper	80 g	Uncoated paper {RER}		
Sheet 3: PE	50 g	LDPE {GLO}		

**Table 5:** Reference inventory and parameter variations resulting from the use of the additive for chicken production.

	Parameters	AGRIBALYSE® Reference	Changes with the Additive
Broiler fattening	Number of chicks–in/Number of broilers–out	155,760/149,179	-
	Age of broilers–out (days)	40	-
	Weight of broilers–out (kg)	1.9	2.0 (+5%)
	Quantity of feed for all animals (kg)	520,175	520,175
	Quantity of CEFA consumed for all animals (kg)	0	130
	ADG (gr/day/animal)	45.7	47.95 (+4%)
	FCR	1.8	1.76 (−3%)
Total broiler production (kg)		281,202	295,263

Reference inventory and parameter variations resulting from the use of the additive for chicken production.

**Table 6:** Reference inventory and parameter variations resulting from the use of the additive for swine production.

	Parameters	AGRIBALYSE^®^ Reference	Changes with the Additive
Post-weaning	Number of piglets–in/Number of pig to be fattened–out	3656/3576	-
	Animal weight–out (kg)	32	-
	Duration (days)	52.5	47.3
	Quantity of feed for all animals (kg)	151,359	136,223
	Quantity of CEFA consumed for all animals (kg)	0	34.1
	ADG (gr/day/animal)	468.6	523.4 (+12%)
	FCR	1.3	1.2 (−7%)
Fattening	Number of fattened pigs–out	3429	-
	Animal weight–out (kg)	115.4	121.0 (+4.53%)
	Duration (days)	108.0	109.4
	Quantity of feed for all animals (kg)	849,504	860,483
	Quantity of Nor-spice AB consumed for all animals (kg)	0	215.1
	ADG (gr/day/animal)	774.4	809.5 (+4.53%)
	FCR	2.1	2.0 (−2.12%)
	Total fattened-pig production (kg)	395,707	413,632

**Table 7:** List of impact categories selected.

Impact Categories	Unit	Main Contributors
Climate change	kg CO_2_ eq	Agriculture, energy use
Land occupation	Pt	Agriculture
Water deprivation	m3 eq	Agriculture, cleaning

## Data Availability

The data that support the findings of this study are available on request from the corresponding author, (H.B.).
